# Chiral Separation of the Phenylglycinol Enantiomers by Stripping Crystallization

**DOI:** 10.3390/molecules23112901

**Published:** 2018-11-07

**Authors:** Lie-Ding Shiau

**Affiliations:** 1 Department of Chemical and Materials Engineering, Chang Gung University, Taoyuan 33302, Taiwan; shiau@mail.cgu.edu.tw; Tel.: +886-3-2118800 (ext. 5291); Fax: +886-3-2118700; 2 Department of Urology, Chang Gung Memorial Hospital, Linkou, Taoyuan 33302, Taiwan

**Keywords:** crystallization, vaporization, purification, phenylglycinol

## Abstract

Stripping crystallization (SC) is introduced in this work for chiral purification of *R*-phenylglycinol from the enantiomer mixture with an initial concentration ranging from 0.90 to 0.97. As opposed to the solid–liquid transformation in melt crystallization, the three-phase transformation occurs in SC at low pressures during the cooling process. SC combines melt crystallization and vaporization to produce a crystalline product and mixture vapor from a mixture melt due to the three-phase transformation. Thermodynamic calculations were applied to determine the operating pressure for the three-phase transformation during the cooling process in the SC experiments. To consider the possible deviations between the calculated and the actual three-phase transformation conditions, the product purity and the recovery ratio of *R*-phenylglycinol were investigated within a range of operating pressures during the cooling process.

## 1. Introduction

Pure enantiomer is often needed for the desired therapeutic effect due to different pharmacological and pharmacokinetic processes for the enantiomers of drugs. However, separation of the enantiomers has long been a challenging task as the enantiomers have nearly identical physical and chemical properties [1]. Various enantioselective separation techniques, including enantioselective synthesis, chromatographic separation, and preferential crystallization, have been developed for chemical and pharmaceutical industries [2]. Preferential crystallization generally has been used as an attractive means to separate the conglomerate-forming enantiomers from racemate [3,4,5,6]. Although chromatographic separation has been investigated extensively [7,8,9,10], the synthesis of efficient chiral stationary phases in chromatographic methods is usually deemed a robust technology. Recently, Didaskalou et al. [11] reported the membrane-grafted asymmetric organocatalyst used as an integrated synthesis–enantioseparation platform. Rukhlenko et al. [12] explored the capabilities of the related enantioseparation method by analytically solving the problem of the force-induced diffusion of chiral nanoparticles in a confined region. 

Phenylglycinol, also called 2-Amino-2-phenylethanol, is an important example of a chiral compound. Only *R*-phenylglycinol can be used as an important precursor of HIV-1 protease inhibitor [13]. Enantioseparation, using extractant impregnated resins [14] or liquid–liquid extraction [15,16] has been proposed to separate *R*-Phenylglycinol from the racemic mixture. Fundamentally, an enantioselective solvent is chosen and used as extractant for the enantioseparation of phenylglycinol.

Stripping crystallization (SC) is a new separation technology, which combines melt crystallization and vaporization to produce a crystalline product due to the three-phase transformation. SC has been successfully developed to separate the mixtures with close boiling temperatures, including mixed xylenes [17,18,19], the styrene/ethylbenzene mixture [20], and the *S*-ibuprofen/*R*-ibuprofen mixture [21]. As opposed to extractant impregnated resins or liquid–liquid extraction, no solvent is added in SC. Thus, no removal of solvent is required at the end of SC. 

The objective of this research is to study the feasibility of SC in purification of *R*-phenylglycinol from a phenylglycinol mixture. The thermodynamic calculations are adopted to determine the three-phase transformation conditions for the SC experiments. The effects of various operating conditions on the enantiomeric purity and recovery ratio of *R*-phenylglycinol crystalline product are investigated

## 2. SC Model

**Figure 1:**
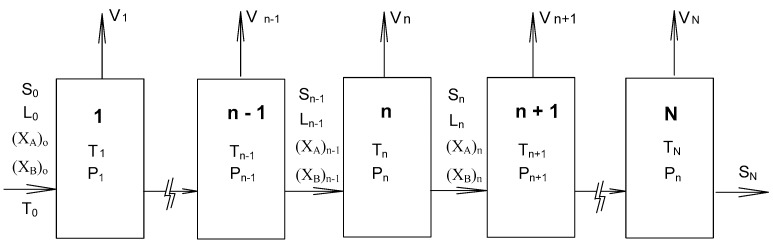
Schematic diagram of the stripping crystallization (SC) operation where each stage is operated at a three-phase transformation state.

As opposed to the solid–liquid transformation in melt crystallization operated at normal pressure during the cooling process [22,23,24,25,26,27], the three-phase transformation occurs in SC at low pressures during the cooling process. Thus, SC combines melt crystallization and vaporization to produce a crystalline product and mixture vapor from a mixture liquid or melt [17,18,19,20,21]. The SC process is simulated in a series of N stage operations shown in Figure 1, where each stage is operated at a three-phase transformation state. The SC process starts with a mixture liquid or melt. The vapor formed in each stage is removed, while the crystalline product and the remaining liquid or melt in each stage enter the next stage. Thus, only the crystalline product remains at the end of SC when the liquid or melt is nearly eliminated.

When SC is applied to purify *R*-phenylglycinol (B-component) from the mixture of *S*-phenylglycinol (A-component) and *R*-phenylglycinol, the SC process starts with a mixture melt of phenylglycinol. The corresponding three-phase transformation condition in each stage is determined based on the following assumptions: (a) The ideal gas law is assumed for the vapor due to low pressures; (b) The ideal solution for the melt is assumed due to the structure similarity between *S*-phenylglycinol and *R*-phenylglycinol; (c) The Clausius–Clapeyron equation [28,29] is adopted to describe the temperature dependence of the saturated pressure for each component in the melt; (d) The sublimation based on the solid–vapor equilibrium is not considered here as the mixture melt is used in the beginning of the experiments. Some physical properties of *S*-phenylglycinol and *R*-phenylglycinol are listed in Table 1. For simplicity, ΔH_V_ = 2ΔH_m_ is assumed in the thermodynamic calculations.

As SC is applied to produce *R*-phenylglycinol crystalline product from a mixture melt due to the three-phase transformation, both the solid–liquid equilibrium and the vapor–liquid equilibrium need to be simultaneously satisfied. The solid–liquid equilibrium is described by the Schroder–Van Laar equation [1,28,29], while the vapor–liquid equilibrium is described by Raoult’s law [28,29]. Consequently, as similar to a previous work reported by Shiau [21], the three-phase equilibrium equations can be derived in each stage. If T_n_ is specified in each stage, these equations can be simultaneously solved for P_n_, (X_A_)_n_, (X_B_)_n_, (Y_A_)_n_ and (Y_B_)_n_ for n = 1, 2, …, N.

**Figure 2:**
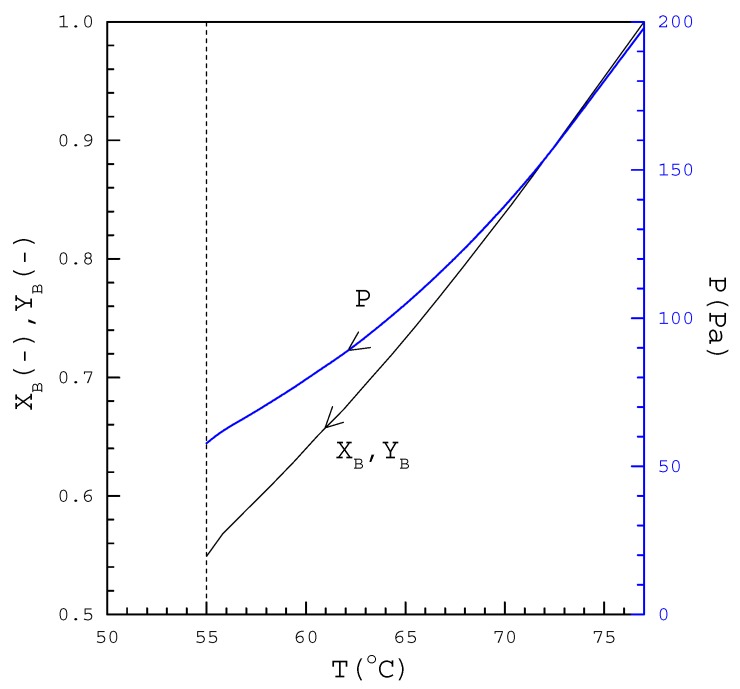
P(T), X_B_(T) and Y_B_(T) based on the thermodynamic calculations for the three-phase transformation.

Figure 2 displays the thermodynamic calculations of P(T), X_B_(T), and Y_B_(T) during the cooling process. Thus, the corresponding pressure, P(T), and the corresponding melt composition of *R*-phenylglycinol, X_B_(T), decreases during the cooling process for SC. In other words, Figure 2 reveals that, as X_B_(T) in a melt decreases, the corresponding temperature and pressure for the three-phase transformation conditions decreases.

As shown in Figure 1, the three-phase transformation occurs in the melt in each stage, leading to the formation of *R*-phenylglycinol crystalline product and mixture vapor. S_n_ and L_n_ represent the amount of *R*-phenylglycinol crystalline product and the melt, respectively, remaining in stage n, while V_n_ represents the amount of the mixture vapor formed and removed in stage n. The entire material balance in stage n can be described by

S_n−1_ + L_n−1_ = S_n_ + L_n_ + V_n_ (1)

where S_n−1_ + L_n−1_ is the total amount of crystalline product and melt entering stage n. As V_n−1_ represents the amount of vapor formed in stage n − 1 that is subsequently removed, it is not part of the equation for stage n. Thus, the amount of melt decreases and the amount of crystalline product increases during the stage operation. 

Although both the melt and the vapor consist of *S*-phenylglycinol and *R*-phenylglycinol, only *R*-phenylglycinol crystalline product is formed in each stage based on the solid–liquid equilibrium described by the Schroder–Van Laar equation [1,28,29]. It is assumed that no impurity trapping occurs in the formation of *R*-phenylglycinol crystalline product based on the thermodynamic calculations. The material balance of *R*-phenylglycinol in stage n can be described by

S_n−1_ + L_n−1_(X_B_)_n−1_ = S_n_ + L_n_(X_B_)_n_ + V_n_(Y_B_)_n_ (2)

It is observed during the experiments that the three-phase transformation occurs in the melt very quickly in each stage, leading to the formation of *R*-phenylglycinol crystalline product and the mixture vapor. Therefore, it is assumed in each stage that the heat released in forming *R*-phenylglycinol crystalline product is quickly removed by vaporizing some portion of the melt. Thus, the energy balance in stage n can be described by

(S_n_ − S_n−1_)ΔH_m,B_ = V_n_ΔH_V,B_ (3)

where S_n_ – S_n−1_ represents the amount of crystalline product formed in stage n while V_n_ represents the amount of melt vaporized in stage n. Note that the heat of vaporization is assumed as ΔH_V,B_ for a mixture melt due to ΔH_V,A_ = ΔH_V,B_.

As only the mixture melt L_0_ with a known (X_B_)_0_ is injected into the sample container, one obtains S_0_ = 0. Equations (1) to (3) can be solved simultaneously for three unknown variables- S_n_, L_n_ and V_n_. Note that S_N_ and L_N_ represents the crystalline product and the melt, respectively, remaining at the end while the total amount vapor formed and removed at the end is given by $\sum_{n=1}^{N}V_{n}$.

## 3. Experimental Section

**Figure 3:**
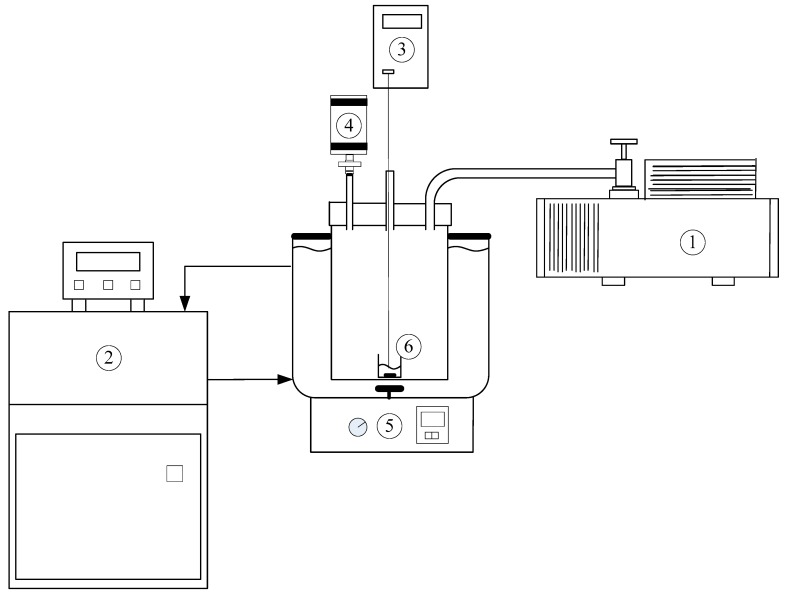
Schematic diagram of the experimental apparatus for the SC operation with the features: (1) Mechanical pump, (2) constant temperature bath, (3) thermocouple, (4) pressure gauge, (5) magnetic stirrer operated at 70 rpm, (6) 5-mL sample container in a 1.5-L chamber.

The experimental assembly consisted of a 5-mL sample container in a 1.5-L chamber as shown in Figure 3. The stainless chamber was immersed in a constant temperature bath. A mechanical vacuum pump was used to lower the pressure in the chamber. A temperature probe was positioned in the center of the mixture melt and a pressure gauge was connected to the big chamber. Thus, the operating temperature and pressure could be adjusted mid-experiment. Crystallization and vaporization of the mixture melt during the three-phase transformation could be observed in the chamber via a transparent cover.

**Figure 4:**
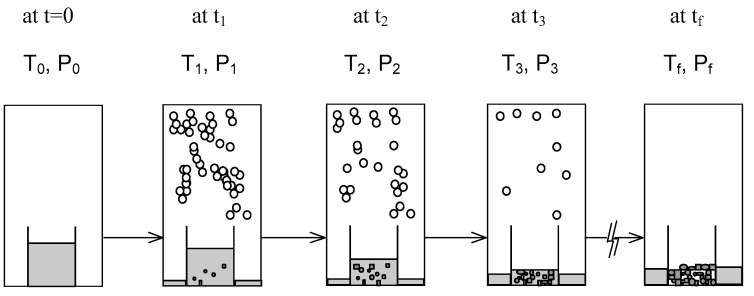
Schematic diagram of a batch SC experiment, where each stage corresponds to a three-phase transformation state at a given time: At t = 0, a mixture melt in the sample container; at 0 < t < t_f_, formation of *R*-phenylglycinol crystalline product and mixture vapor from a mixture melt due to the three-phase transformation; at t_f_, only *R*-phenylglycinol crystalline product and the remaining melt left in the sample container. Note that the vapor was condensed and collected outside the sample container in the chamber.

*R*-phenylglycinol (purity >98%) and *S*-phenylglycinol (purity >98%) were purchased from ACROS. In the beginning of the experiment, 1 g mixture melt with a known concentration was injected into the sample container stirred by a magnetic bar at 70 rpm. Then, the temperature was lowered gradually from the melting point (77 °C). The cooling rate generally started at 0.5 °C/min in the beginning and then increased gradually to 1 °C/min in the later stage. As the temperature decreased, pressure was adjusted downward based on Figure 2. Thus, a series of three-phase transformations occurred in the melt, leading to the formation of *R*-phenylglycinol crystalline product and mixture vapor. The experiments were generally ended at around 55 °C and 58 Pa within 25 min when vaporization was no longer observed in the chamber. Figure 4 illustrates the schematic diagram of the batch experiments, in which the melt was simultaneously vaporized and crystallized due to the three-phase transformation. Upon completion, the final product, including the crystals and melt, in the sample container were weighed.

The enantiomeric purity of the final product was analyzed by Polarimeter (Horiba, model: SEPA-300). The polarimetry was measured by dissolving 0.1 g final product in 20 mL 1 M HCl solution. First, a plot of the measured specific optical rotation versus the known enantiomeric purity within the range X_B,0_ = 0.9 to 1.0 was fitted with a linear regression line. Then, by measuring the specific optical rotation of the final sample the enantiomeric purity could be determined. Note that ${\left[\alpha \right]}_{D}^{20}$ = −29.9° for *R*-phenylglycinol and ${\left[\alpha \right]}_{D}^{20}$ = 29.9° for *S*-phenylglycinol. It should be noted that, as only crystallization and vaporization occurred during SC, polarimetry could be used to determine the enantiomeric purity of the final product.

From a practical point of view, some solvent might remain in the mixture melt before SC. To elucidate the effects of residual solvent on the final product purity and recovery ratio of *R*-phenylglycinol, 0.1 g ethanol was added into 1 g mixture melt in the beginning of the SC experiments. It was found that the final product purity and recovery ratio for 1 g mixture melt with 0.1 g ethanol were nearly the same as those for 1 g mixture melt without ethanol. Thus, all ethanol was vaporized when SC was operated at low pressures during the cooling process. Solvent inclusion in the formation of *R*-phenylglycinol crystalline product was nearly negligible.

## 4. Results and Discussion

SC was applied to purify *R*-phenylglycinol for various 1 g feeds: Feed 1 with (X_B_)_0_ = 0.90, feed 2 with (X_B_)_0_ = 0.95, and feed 3 with (X_B_)_0_ = 0.97. Table 2 lists the thermodynamic calculations for 1 g feed 1, where T_0_ = 72.7 °C is the initial three-phase transformation temperature for the mixture melt. As vaporization was no longer observed in the experiments at around 55 °C, T_N_ = 54.6 °C was chosen for N = 15 with ΔT = 1.2 °C. Thus, T_n_ was specified in each stage for n = 1, 2, …, N using T_n−1_ − T_n_ = ΔT. P_n_, (X_A_)_n_, (X_B_)_n_, (Y_A_)_n_, and (Y_B_)_n_ were determined in each stage by solving the thermodynamic equations while S_n_, L_n_, and V_n_ were determined in each stage by solving Equations (1) to (3) for L_0_ = 1 g and S_0_ = 0. Note that P_n_, (X_B_)_n_ and (Y_B_)_n_ in Table 2 were consistent with the results shown in Figure 2. Table 2 also indicates that, as n increased during the cooling process, S_n_ increased and L_n_ decreased. As SC was operated from 73 °C and 160 Pa (n = 1) to 55 °C and 58 Pa, (n = 15), only *R*-phenylglycinol crystalline product remain in the last stage (S_N_ = 0.606 g) while the melt was nearly eliminated in the last stage L_N_ = 0.098 g. Similar calculated results were obtained for feed 2 and feed 3.

The calculated purity of *R*-phenylglycinol in the final product, including the final crystalline product and the remaining melt, is defined as

(4) $$X_{B,C}=\frac{S_{N}+L_{N}{\left(X_{B}\right)}_{N}}{S_{N}+L_{N}}$$

where S_N_, L_N_ and (X_B_)_N_ are determined in the last stage based on the thermodynamic calculations. The calculated recovery ratio of *R*-phenylglycinol is defined as

(5) $$R_{C}=\frac{S_{N}+L_{N}{\left(X_{B}\right)}_{N}}{L_{0}X_{B,0}}$$

where L_0_ is the initial weight of the mixture melt and X_B,0_ denotes the initial purity of *R*-phenylglycinol in the mixture melt. For example, as shown in Table 2, feed 1 yields S_N_ = 0.606 g and L_N_ = 0.098 g with (X_B_)_N_ = 0.549 in the last stage (N = 15), leading to X_B,C_ = 0.937 and R_C_ = 73% using Equations (4) to (5).

The experimental recovery ratio of *R*-phenylglycinol is defined as

(6) $$R_{f}=\frac{W_{f}X_{B,f}}{L_{0}X_{B,0}}$$

where W_f_ refers to the final weight of the product including the crystalline product and the remaining melt obtained at the end of the experiment, and X_B,f_ represents the experimental purity of *R*-phenylglycinol in the final product.

**Figure 5:**
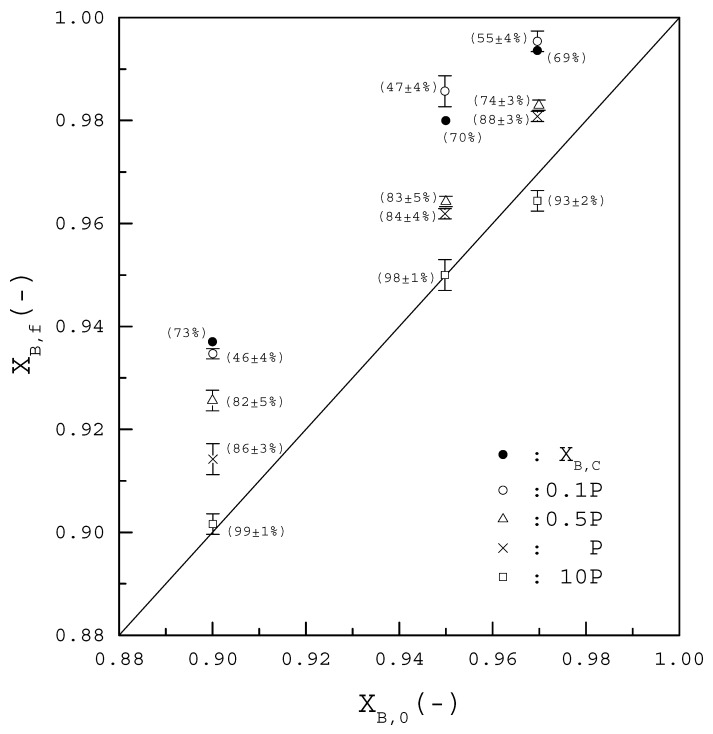
X_B,f_ of the final product plotted against X_B,0_ of the initial feed for various 1 g feeds. The solid line represents X_B,f_ = X_B,0_ indicating no further purification for the initial feed during SC. Solid circle represents the calculated X_B,C_ and the number in parenthesis represents the calculated R_C_. Other symbols, including open circle, open triangle, cross sign, and open square, represent the average of the experimental X_B,f_ for four repetitive experiments operated at the specified pressure and the error bar represents the 95% confidence interval for the experimental X_B,f_. The number in parenthesis represents the average of the experimental R_f_ with the 95% confidence interval for the experimental R_f_. Note that no error bar is added for solid circle of the calculated X_B,C_.

Figure 5 shows X_B,f_ of the final product plotted against X_B,0_ of the initial feed for various feeds. The Solid circles represent the calculated X_B,C_ and the number in parenthesis represents the calculated R_C_. Thus, the thermodynamic calculations predict that feed 1 can be purified from X_B,0_ = 0.90 to X_B,C_ = 0.937 with R_C_ = 73%, feed 2 can be purified from X_B,0_ = 0.95 to X_B,C_ = 0.979 with R_C_ = 70%, and feed 3 can be purified from X_B,0_ = 0.97 to X_B,C_ = 0.995 with R_C_ = 69%.

Other symbols in Figure 5, including open circle, open triangle, cross sign, and open square, represent the average of the experimental X_B,f_ for four repetitive experiments operated at the specified pressure and the error bar represents the 95% confidence interval for the experimental X_B,f_. The number in parenthesis represents the average of the experimental R_f_ with the 95% confidence interval for the experimental R_f_. For example, cross sign represents the average X_B,f_ when the operating pressures was controlled at P(T) during the cooling process. As a lower X_B,f_ with a higher R_f_ was observed for each feed compared to the calculated X_B,C_ and R_C_, it was speculated that the calculated pressure P(T) in Figure 3 might be higher than the actual three-phase transformation pressure, leading to less impurity (*S*-phenylglycinol) vaporized and more crystalline product formed from the melt during the cooling process.

To consider the possible deviations between the calculated and the actual three-phase transformation pressure, various operating pressures are compared during the cooling process. The open circle in Figure 5 represents the average X_B,f_ when the operating pressures were controlled at 0.1 × P(T) during the cooling process. Similarly, the open triangle represents the average X_B,f_ when the operating pressures were controlled at 0.5 × P(T). The open square represents the average X_B,f_ when the operating pressures were controlled at 10 × P(T).

Figure 5 shows for each feed that X_B,f_ increased with decreasing pressure while R_f_ decreased with decreasing pressure. For example, when SC was applied for X_B,0_ = 0.90, X_B,f_ increased from 0.914 to 0.935 and R_f_ decreased from 86% to 46% as the operating pressure was decreased from P(T) to 0.1 × P(T). As shown in the figure, when 0.1 × P(T) was adopted for each X_B,0_, X_B,f_ was close to X_B,C_ with R_f_ (46% to 55%) < R_C_ (69% to 73%). Consequently, compared to the calculated P(T) in Figure 3, 0.1 × P(T) should be closer to the actual the three-phase transformation pressure. On the other hand, when 10 × P(T) was adopted for each X_B,0_, X_B,f_ was close to X_B,0_ with R_f_ = 93% to 99%, indicating that the feed was not further purified in the SC experiments.

Discrepancies between the thermodynamic calculations and the experimental results are attributed to (a) the assumption that each stage was operated at the three-phase transformation. However, experimentally, these might not always be achieved; (b) although pure *S*-phenylglycinol crystal should be formed based on the thermodynamic equilibrium, impurity trapping can occur under actual kinetic conditions. The scope of this work was to investigate the feasibility of SC in the purification of *R*-phenylglycinol from a phenylglycinol mixture. In future kinetic studies, the effects of process conditions (e.g., cooling rate) on the crystal growth kinetics and impurity inclusion will be explored based on the impurity trapping correlation proposed by Myerson and Kirwan [31,32].

## 5. Conclusions

SC was successfully applied for chiral purification of *R*-phenylglycinol from the phenylglycinol enantiomers. A lower pressure during the cooling process generally led to a higher experimental product purity with a lower experimental recovery ratio. When SC was operated under the optimal pressure, which was one-tenth of the pressure based on the thermodynamic calculations, the experimental product purity was close to the calculated product purity while the experimental recovery ratio was slightly lower than the calculated recovery ratio. In other words, when temperature and pressure was lowered from 72.7 °C and 15 Pa to 55 °C and 6 Pa during SC, the purity of *R*-phenylglycinol increased from 0.90 to 0.937, from 0.94 to 0.985, and from 0.97 to 0.995 respectively with the recovery ratio ranging between 46% to 55%.

As no solvent is added into the melt, SC is a clean separation technology. Compared to melt crystallization, neither solid/liquid separation nor crystal washing is required because no mother liquor adheres to the crystal surfaces upon completion. Although a portion of the phenylglycinol enantiomers is lost through the vapor stream of each stage, the vaporized mixture can be recycled for continuous operation or mixed with the feed in the next batch for batch operation. The major difficulty in application of SC lies in the required low pressures during the cooling process. Furthermore, the crystal growth kinetics and impurity trapping during SC need to be elucidated in order to design an apparatus for industrial application.

## Figures and Tables

**Table 1:** Some physical properties for phenylglycinol.

Property	Phenylglycinol
molecular structure	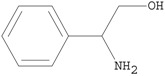
molecular weight	137.2
melting point ^a^, °C	77
boiling point ^a^, °C	261
triple-point pressure ^b^, Pa (N/m^2^)	198
heat of melting ^c^, J/mol	2.57 × 10^4^

^a^: The Merck Index [30]; ^b^: Estimated by Clausius–Clapeyron equation [28,29]; ^c^: Measured by Differential scanning calorimetry in this work.

**Table 2:** The thermodynamic calculations for 1 g feed with X_B,0_ = 0.90 (ΔT = 1.2 °C).

n	T (°C)	P (Pa)	L (g)	S (g)	V (g)	X_B_
0	72.7	159.4	1	0	0	0.90
1	71.5	149.5	0.672	0.219	0.109	0.872
2	70.3	140.1	0.496	0.336	0.059	0.845
3	69.1	131.3	0.389	0.407	0.036	0.819
4	67.9	122.9	0.316	0.456	0.024	0.793
5	66.7	115.1	0.265	0.490	0.017	0.768
6	65.5	107.6	0.226	0.516	0.013	0.743
7	64.3	100.7	0.197	0.535	0.010	0.719
8	63.1	94.1	0.175	0.550	0.007	0.696
9	61.9	87.9	0.156	0.562	0.006	0.673
10	60.6	82.1	0.142	0.572	0.005	0.651
11	59.4	76.6	0.129	0.581	0.004	0.629
12	58.2	71.5	0.119	0.588	0.003	0.608
13	57.0	66.6	0.110	0.593	0.003	0.588
14	55.8	62.1	0.102	0.598	0.003	0.568
15	54.6	57.8	0.096	0.603	0.002	0.549

## Notation

ΔH_m,i_	heat of melting for component-i (>0), J/mol
ΔH_V,i_	heat of vaporization for component-i (>0), J/mol
L_n_	mass of the liquid phase out of stage, n, g
N	an integer number (>2), dimensionless
P	pressure, Pa
R_C_	calculated recovery ratio, dimensionless
R_f_	experimental recovery ratio, dimensionless
S_n_	mass of the solid phase out of stage, n, g
T	boiling temperature of component-i, K
T_b.i_	boiling temperature of component-i, K
T_m.i_	melting temperature of component-i, K
T_tri.i_	triple-point temperature of component-i, K
V_n_	mass of the vapor phase out of stage, n, g
X_i_	mole fraction of component-i in melt, dimensionless
Y_i_	mole fraction of component-i in vapor phase, dimensionless

## Subscript

0	in the initial feed
n	in stage, n
N	in the last stage
